# Behaviour of Solitary Adult Scandinavian Brown Bears (*Ursus arctos*) when Approached by Humans on Foot

**DOI:** 10.1371/journal.pone.0031699

**Published:** 2012-02-20

**Authors:** Gro Kvelprud Moen, Ole-Gunnar Støen, Veronica Sahlén, Jon E. Swenson

**Affiliations:** 1 Department of Ecology and Natural Resource Management, Norwegian University of Life Sciences, Ås, Norway; 2 Department of Wildlife, Fish and Environmental Studies, Swedish University of Agricultural Sciences, Umeå, Sweden; 3 Norwegian Institute for Nature Research, Trondheim, Norway; Phillip Island Nature Parks, Australia

## Abstract

Successful management has brought the Scandinavian brown bear (*Ursus arctos* L.) back from the brink of extinction, but as the population grows and expands the probability of bear-human encounters increases. More people express concerns about spending time in the forest, because of the possibility of encountering bears, and acceptance for the bear is decreasing. In this context, reliable information about the bear's normal behaviour during bear-human encounters is important. Here we describe the behaviour of brown bears when encountering humans on foot. During 2006–2009, we approached 30 adult (21 females, 9 males) GPS-collared bears 169 times during midday, using 1-minute positioning before, during and after the approach. Observer movements were registered with a handheld GPS. The approaches started 869±348 m from the bears, with the wind towards the bear when passing it at approximately 50 m. The bears were detected in 15% of the approaches, and none of the bears displayed any aggressive behaviour. Most bears (80%) left the initial site during the approach, going away from the observers, whereas some remained at the initial site after being approached (20%). Young bears left more often than older bears, possibly due to differences in experience, but the difference between ages decreased during the berry season compared to the pre-berry season. The flight initiation distance was longer for active bears (115±94 m) than passive bears (69±47 m), and was further affected by horizontal vegetation cover and the bear's age. Our findings show that bears try to avoid confrontations with humans on foot, and support the conclusions of earlier studies that the Scandinavian brown bear is normally not aggressive during encounters with humans.

## Introduction

Human disturbance can influence wildlife negatively by e.g. preventing successful breeding [1], [2], causing animals to avoid quality foraging areas or quality habitats [3]–[6], altering activity patterns [7], [8], or distribution patterns [9], [10], or even causing increased mortality [11]. Carnivores often present a special challenge to managers, due to the negative attitudes associated with carnivore-human conflicts, e.g. killing livestock, threats to human life and challenges regarding reintroduction [12]–[15]. Bears (*Ursus* spp.) are commonly associated with undisturbed areas away from high human densities. Human disturbance can cause grizzly bears (*Ursus arctos* L.) to use less productive habitats [16]–[18] and habitats with low levels of human use [19]. The Scandinavian brown bear tends to avoid habitats close to forest roads, cabin resorts, and towns [20], [21] and brown bears in Finland have been displaced from previously used habitat by large-scale mechanised forestry [22].

The introduction of bounties in Sweden (1647) and Norway (1733), and the subsequent intensive hunting [23], [24], reduced the Scandinavian brown bear population from 4,000–5,000 individuals in the 1850's to approximately 130 animals around 1930 [25]. Brown bears received protection in Sweden in 1927 and in Norway in 1973, however the Norwegian population was functionally extinct by 1931 [25]. After a slow recovery, the Scandinavian population consisted of around 700 individuals in 1995 [25]. The latest estimates are about 3,300 individuals in Sweden [26] and a minimum of 166 individuals in Norway [27]. Whereas the brown bear population has increased in size and distribution, the areas undisturbed by humans have decreased rapidly. An expanding bear population and extending human activities into the remaining habitats will most likely lead to more frequent bear-human encounters. In fact, there has been an increase in bear-caused human injuries since 1977, especially for hunters, and two people have been killed (O.-G. Støen et al. unpublished). In 2006, a bear-caused human fatality was documented in Finland, the first one since 1936 [28]. The incidents in Sweden have received high media attention and may have contributed to a documented reduction in Swedish people's tolerance towards bears [29]. This reduction in tolerance is more prominent in counties with carnivore presence than the rest of the country. People in Norway are also more afraid of brown bears and wolves (*Canis lupus* L.) than of the two other large carnivores in the country, Eurasian lynx (*Lynx lynx* L.) and wolverine (*Gulo gulo* L.) [30]. However, bear aggressiveness varies geographically and the brown bear in Scandinavia appears to be less aggressive than those in Russia and North America, and only truly dangerous when wounded [31].

The management challenges of the increasing brown bear population include not only people's fear of carnivores in general, but also fear of the unknown [32]. Informing people about the biology and normal behaviour of large carnivores is a good management strategy to reduce people's fear [33] and increase public acceptance. This is essential to maintain sufficient population sizes in areas where carnivores already are present, as well as a requirement for a successful reintroduction of bears [34]. Although most of the bear-injured people were hunters, there are many more hikers and other recreational users in Scandinavian forests, where the public has the right of trespass on private lands. With increased numbers of bear-injured people and declining acceptance of bears, it is important to document how brown bears normally behave when approached by humans.

In this study, we have used technology that allowed us to determine the behaviour of the bears when encountering humans on foot without observing the bears in the field. Our main goals in this study are 1) to describe how solitary adult bears react to human approaches and 2) to identify factors affecting how bears react to human encounters. This knowledge can help managers when giving advice about what people in Scandinavia can expect when walking in areas with brown bears.

## Materials and Methods

### Study area

This study was conducted in the southernmost reproduction area of the Scandinavian brown bear population in Sweden (61°N, 14°E). The area consists of gently rolling hills, and most of the area (>90%) lies below the timberline (∼750 m a.s.l.) [35]. The forest is heavily managed and dominated by Scots pine (*Pinus sylvestris* L.) and Norway spruce (*Picea abies* H. Karst). About 8% of the forested areas are clear-cuts, and about 40% of the forest is younger than 35 years [36]. The area is sparsely populated by humans, but there is an extensive road system, consisting of small gravel roads and paved public roads [21]. The bear population in the area is hunted and the density is about 30 individuals per 1000 km^2^ [37], [38].

### The bears

We approached 21 female and 9 male radio-collared solitary adult bears; 4 to 19 years old. Of these, 14 females and 3 males were approached in more than one year. The bears were equipped with GPS Plus-3 or GPS Pro-4 neck collars (VECTRONIC Aerospace GmbH, Berlin, Germany), and a VHF transmitter implant (IMP 400L) (Telonics, USA). Methods used for marking and capturing bears have been described earlier [39], [40]. All the bears used in this study were captured and handled in March - May the year of their respective approaches, i.e. 1–4 months prior to the start of the approach experiments. Bears can be captured for the first time both as adults and subadults, and older bears have therefore not necessarily been handled more often than younger bears. Bears in the study area reach 90% of their adult size at 4.1 years of age, and we defined the bears as adult when 4 years or older [41]. If the bear was not followed from birth, the age was determined by counting annuli of a cross-section of one of the premolar roots [42]. The bears were approached a maximum of six times each year, and we waited at least fourteen days between each approach of the same individual. The Scandinavian brown bear population is hunted, and the annual brown bear hunting season in Sweden starts on 21 August and ends on 15 October or when quotas are filled. The capturing of the bears were approved by the Swedish Environmental Protection Agency (permit Dnr 412-7327-09 Nv) and the approaches were approved by the appropriate ethical committee i.e. Djuretiska nämnden in Uppsala, Sweden (permit C 47/9).

### The approaches

We conducted 169 approaches; 19 in 2006 (29 June to 14 August), 61 in 2007 (7 June to 4 October), 76 in 2008 (6 June to 24 October), and 13 in 2009 (13 August to 10 October). We divided the field seasons into a pre-berry season (spring/early summer) and a berry season (summer/autumn), because the bears could potentially change behaviour after entering the period of hyperphagia in late summer. We used the date when we first observed fresh berries in the scats to separate the seasons; 20 July in 2006, 13 July in 2007, 14 July in 2008. In 2009, all the approaches were conducted in the berry season. Before an approach, we programmed the collars to register a GPS position every minute for three hours. Programming of the collars was made via a web-based SMS scheduling service approximately a week before the approach. Of the theoretical maximum of 181 GPS positions per bear per approach, we received 66±21 (mean ± SD) positions (37±12% of theoretical maximum) in 2006, 89±30 positions (47±16%) in 2007, 145±43 positions (80±24%) in 2008, and 177±3 positions (98±1%) in 2009. The increasing proportion of the theoretical maximum of positions received over the years was probably due to improved quality of the GPS collars, with increased position accuracy and fewer erroneous positions (Robert Schulte, Vectronic GmbH, pers.comm). The positions were stored, sent to a base station via SMS, and downloaded to a computer. The approaches started after one hour of 1-minute positions, between 11:00 hrs and 16:00 hrs local time. This time of the day was chosen because the bears are usually inactive in a resting site at this time [43], and because this is the time when most people are in the forest.

Prior to the approach, the bears were located using triangulations of the VHF signals from the radio collar and/or the implant using a portable receiver, a roof-mounted omni-directional antenna, and a hand-held yagi-antenna. One to four people, hereafter referred to as the observers, conducted the approaches. During the approach, the bear was monitored with VHF-tracking equipment, which enabled the observers to monitor the bear's movements while passing close by. The approaches started 869±348 m (n = 154) from the bear, and were directed so that the observers would pass the bear upwind of it, with the wind coming at a 90° angle, and at a distance of approximately 50 m. The wind strength was measured when passing the initial site using the Beaufort Wind Scale (scale from 1 (1–3 mph) to 12 (73+ mph)). The observers continued for 500 m, and then walked back to the starting point with a minimum distance of 500 m from the bear's original location. The observers talked with each other and kept a normal hiking pace of 3.4±0.6 km/h (minimum 2.1 km/h, maximum 5.1 km/h). When just one observer approached the bear, this person talked to him- or herself. During the approach, the track of the observers was registered with a hand-held GPS receiver (Garmin GPSMAP 60CSx (Garmin Ltd., USA) or Magellan SporTrack Color (Thales, Santa Clara, California, USA)) that was programmed to record positions every 10 m. After the approach, the observer's tracklog was downloaded into the computer.

### Passive and active bears

Based on the GPS positions from the start of the 1-minute positioning to the start of the approach, hereafter referred to as the control period, we could recognise two behaviours, passive and active. The bear was regarded as passive if it remained within a limited area that had a diameter between the outer GPS positions <70 m (30 m±13 m, minimum 8 m, maximum 69 m), hereafter referred to as a cluster. Passive bears were usually resting, and we usually found daybeds in the cluster. The bear was regarded as active if the positions indicated movement. The distance between the two outermost positions were on average 411±327 m (minimum 85 m, maximum 1092 m), and active bears were usually foraging. Depending on behaviour, as described above, the bears were grouped into passive and active for analysis. Most bears were either active or passive during the whole period, but 14 bears were active during the control period and became passive just before the approach started and were therefore analysed as passive bears. Eight bears were passive and became active during the control period, and were therefore analysed as active bears.

### Habitat description

One to 41 days (median 4 days) after the approach, field personnel visited the clusters and described the vegetation where the bear had stayed during the control period, hereafter referred to as the initial site, and the cluster where the bear settled down after being disturbed, hereafter referred to as the second site. In cases where the bear was active during the control period, the last GPS position from the bear during the control period was defined as the initial site. We searched for daybeds, excrements, and other bear signs at the sites. In 2006, the horizontal vegetation cover in the initial and the second site was measured with an umbrella that was 95 cm in diameter and divided into eight equal sectors. The horizontal vegetation cover was measured at 10 m in every cardinal direction, and the sectors were scored for visibility (0 = 0–33% visibility, 0.5 = 33–66% visibility and 1 = 66–100%) with a maximum score of 32 if fully visible.The sums of the scores were used in the analyses. In 2007 to 2009, we measured the horizontal vegetation cover in the initial and at the second site as the sighting distance with a cylinder; 60 cm tall and 30 cm in diameter. This cylinder was divided into 2 colours, a red upper part and a white lower part [44]. We placed the cylinder in the bed, or in the mid-point of the initial site/second site when no bed was found, and walked in the cardinal directions until we no longer could see the cylinder.

To use the horizontal vegetation cover data from 2006, we estimated the comparability of the two sampling methods by using both the umbrella and the cylinder in 53 plots in 2007. The sum of the umbrella score in all cardinal directions (Sum_UMBRELLA_) was regressed on the average of the distances in the four cardinal directions using the cylinder sighting distance (Average_CYLINDER_). The linear equation was Average_CYLINDER_ = 10.7+(0.73*Sum_UMBRELLA_). The regression analyses showed a linear relationship (R^2^adj = 53.7%, n = 53, p<0.000). For the analyses, we used the estimated sighting distance from this equation for 2006, and the observed sighting distance for 2007, 2008 and 2009.

### Data analysis

We did not find any difference in the maximum distance bears moved between the first and the second hour of 1-minute positions for bears that had been scheduled for an approach, but were not approached (two-tailed t-test: t_21_ = 0.28 , p = 0.78, n = 22). Hence, we assumed that the bears would behave similarly in the control period and the following hour if they had not been disturbed. We calculated the speed between two successive positions (m/min), and transformed the data by (log(speed*100)) to normalise the residual distribution. Using statistical quality control, we estimated an upper control limit (UCL) [45] for the speed between two positions for passive and active bears during the control period. Only data from bears that stayed passive or active during the entire control period were used in the calculations of UCL. Based on UCL, we judged that passive and active bears had been disturbed once they reached speeds above 33.5 m/min (2.01 km/h) and 101.3 m/min (6.08 km/h), respectively.

If the bear remained in its initial site while being approached, we defined the tolerance distance as the shortest distance to the passing observers. When the speed between two positions exceeded the behaviour-specific UCL, we used ESRI® ArcMap™ 9.2 [46] to determine if this reaction occurred before or after the observers passed the bear. The distance to the observer at the time of the reaction was defined as the flight initiation distance (FID) [47]–[52]. When calculating FID, we did not include approaches where more than one GPS position from the bear was missing around the time of disturbance. The GPS position prior to the GPS position exceeding UCL was defined as the FID, and hence used for the calculation of the distances to the observers. In 15 approaches, the bears left the initial site, but the speed in the movement did not exceed UCL and we could therefore not determine FID. In four approaches, the bears left the site after the observers had ended their approach, and FIDs were not determined.

After leaving the initial site, some of the bears settled in a second site before the 1-minute positioning period was over. The distance between the coordinates of the beds in the initial and the second site was defined as the distance moved. At sites where a bed was found, but no coordinates were registered by field personnel, the midpoint of the cluster was used as the position of the site (n = 27). For active bears, we used the GPS position of FID as the start to determine the distance moved. We defined the time the bear spent active after disturbance as the time interval in minutes from the GPS position of FID to the first position in the second site.

We used generalised linear mixed models to determine if various variables were related to whether the bears remained or moved (using binomial link function), and linear mixed models for the analysis of the FID. The initial models consisted of the following variables and interactions: Age of the bear; Sex of the bear; Cover (sighting distance in the initial site); Activity of the bear (passive = 0; active = 1); Season (pre-berry = 0; berry = 1); Minimum distance between observer and initial site (only in the binomial model); Carcass present at initial site; Wind strength near bear; Number of observers; Age of the bear*Cover; Age of the bear*Activity of the bear; Age of the bear*Season; Sex of the bear*Cover; Sex of the bear*Activity of the bear; Sex of the bear*Season; Cover* Activity of the bear; Cover*Season. An AIC-based backward elimination was performed on these models and the final models were selected based on the lowest value of AIC [53] (Table S1). We chose mixed models in order to account for the random effect of each individual bear using Bear ID as a random effect in the models, and thereby avoid biases caused by pseudoreplication. We used the statistical programming language and environment R version 2.8.1 [54], and the lmer (lme4 library) package.

## Results

We passed the bears' initial sites at an average of 54±61 m (n = 131), which was further than the average sighting distance in the initial sites (18±7 m, n = 120). There was significantly less cover in initial sites (25±10 m, n = 21) than second sites (17±8 m, n = 21) for active bears (two-tailed paired t-test: t_31_ = 2.88, p = 0.007), but no difference between the initial sites (17±8 m, n = 99) and second sites (16±6 m, n = 95) for passive bears (two-tailed paired t-test: t_183_ = 1.07, p = 0.29). The initial site of active bears had significantly less cover than those of passive bears (two-tailed t-test: t_22_ = −3.80, p = 0.001), but there was no difference in cover in the second sites of passive and active bears (two-tailed paired t-test: t_29_ = −0.65, p = 0.52).

### Detection of the bears

None of the bears displayed any aggressive behaviour towards the observers, and none of the observers reported feeling threatened during any of the approaches. Bears were detected in 15% of the approaches (n = 154); 17 bears were seen, we heard movements from five bears, and during one approach we heard vocalization and movements. The detection rate did not vary with the sex of the bear (chi-squared test: χ^2^ = 0.82, df = 1, p = 0.36), or the season (chi-squared test: χ^2^ = 0.38, df = 1, p = 0.54). Most of the 17 bears were first seen while standing still, and after the initial observation, all of the bears walked or ran away. We observed a fresh carcass in eight of the initial sites.

### Remaining or moving

The bears left the initial site and moved away from the observers in 80% of the approaches (n = 148); the bears that remained had a tolerance distance of 84±64 m (median 62 m, minimum 23 m, maximum 313 m, n = 30). The older bears remained more often than the younger bears, but this difference decreased during the berry season (Table 1). We also found a tendency for the bears to leave more often with increasing number of observers (Table 1). The other variables were not related to whether the bears remained or left their initial site (Table 1).

**Table 1:** Results from the generalised linear mixed model for remaining or leaving the initial site.

Explanatory variables	β	SE	Z	P
Age of the bear	−0.558	0.223	−2.503	0.012
Sex of the bear (male = 0, female = 1)	−2.769	1.769	−1.536	0.125
Cover (sighting distance at the initial site)	0.204	0.215	0.945	0.345
Season (pre-berry = 0, berry = 1)	0.860	1.866	0.461	0.645
Wind strength near bear	−0.313	0.277	−1.128	0.259
Number of observers	0.843	0.488	1.727	0.084
Age of the bear * Season	0.304	0.130	2.338	0.019
Sex of the bear * Cover	0.128	0.099	1.288	0.198
Cover * Season	−0.139	0.108	−1.292	0.197

Results from the generalised linear mixed model (binomial link function) explaining whether brown bears remained (0) or left (1) their initial site when approached by humans on foot in central Sweden in 2006–2009 (n = 148). Test statistics are given for the model with the lowest value of AIC. The parameter *β* is the slope, SE denotes the standard error, Z denotes the z-value, and P denotes the p-value for the test.

### Flight initiation distance (FID)

**Figure 1:**
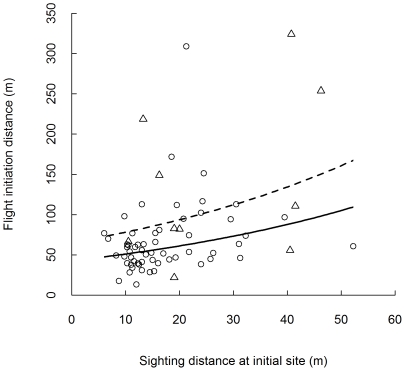
Flight initiation distance (FID) in relation to sighting distance at the initial site. Distribution of flight initiation distance (FID) for passive (circles and full line) and active (triangles and broken line) Scandinavian brown bears approached by humans on foot in central Sweden in 2006–2009 (n = 78), in relation to sighting distance at the initial site (shorter sighting distance indicates more horizontal vegetation cover).

**Figure 2:**
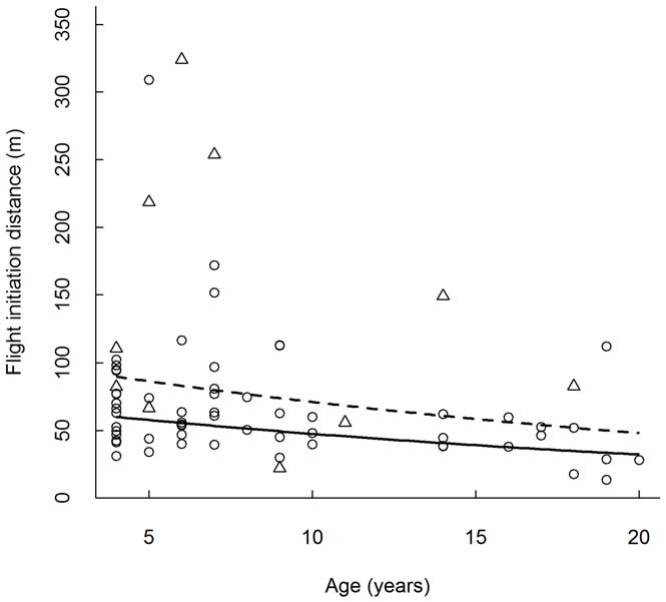
Flight initiation distance (FID) in relation to age of the bear. Distribution of flight initiation distance (FID) for passive (circles and full line) and active (triangles and broken line) Scandinavian brown bears approached by humans on foot in central Sweden in 2006–2009 (n = 78), in relation to the age of the bear.

Passive bears that left before we passed the initial site had an average FID of 69±47 m (median 59.6 m, minimum 13 m, maximum 309 m, n = 65). Nine passive bears that remained at their initial site when we passed them at an average distance of 68±68 m (median 159 m, minimum 27 m, maximum 248 m) left when the observers were on average 326±356 m (minimum 68 m, maximum 1221 m) away. Active bears that left before we passed them had an average FID of 115±94 m (median 82.3 m, minimum 22 m, maximum 324 m, n = 13). The bears that left before we passed the initial site left at a shorter distance when there was more horizontal vegetative cover at the initial site (Table 2, Fig. 1). Younger bears left at a longer distance than older bears, and passive bears left at a shorter distance than active bears (Table 2, Figs. 1 and 2). The other variables did not seem to affect FID.

**Table 2:** Results from the linear mixed model for flight initiation distance (FID).

Explanatory variables	β	SE	T
Age of the bear	−0.039	0.013	−3.038
Cover (Sighting distance at the initial site)	0.018	0.007	2.655
Activity of the bear (passive = 0, active = 1)	0.410	0.187	2.189

Results from the linear mixed model (Gaussian link function) explaining the flight initiation distance (FID) for brown bears when approached by humans on foot in central Sweden in 2006–2009 (n = 78). Test statistics are given for the model with the lowest value of AIC. The parameter *β* is the slope, SE denotes the standard error and T denotes the t-value.

### Distance moved and time spent active

The bears that settled at a new site after leaving their initial site before the schedule with 1-minute positions ended were active for 24±23 min (minimum 2 min, maximum 101 min, n = 78), and moved on average 1,173±1,094 m (minimum 99 m, maximum 6,291 m, n = 92) before they settled at the second site. Neither activity behaviour, age of the bear, season, the closest distance between observer and bear, nor sex of the bear was related to the time they spent active or the distance they moved (all p-values>0.22).

## Discussion

### Detection of the bears

None of the approached bears showed any form of aggressive behaviour, which is consistent with the view that the Scandinavian brown bear is less aggressive than brown bears in Asia and North America [31]. This may be a result of the extermination attempt during the 1600–1800's, when bold animals may have been removed selectively [25], [31]. The few brown bears that survived in Sweden around the 1930s were reported to be wary [55]. The present population may contain more bold individuals because the population is larger; however hunting might take out some of the bolder individuals first [56]. The Scandinavian brown bear can act aggressively if wounded, when with cubs of the year, when surprised at carcasses, or if hunting dogs are involved in the encounter [31]. However, the bears we approached near carcasses did not show any aggressive behaviour. Most bears were standing still when first observed and changed behaviour after being detected; by walking or running away. This strengthens our conclusion that the bears wanted to avoid confrontations with humans.

We detected the bears in only 15% of the approaches. This is a low proportion considering that the observers knew the direction and the approximate distance to the bear. This clearly indicates that most encounters between hikers and bears go unnoticed by humans. This could be because bears tend to use densely vegetated sites as their daybed sites [43]. After the encounters, both active and passive bears settled in densely vegetated sites, perhaps to avoid exposing themselves to humans. The fact that there was no difference in sighting distances between initial and second sites of passive bears shows that the bears always select quite dense resting areas. Active bears were disturbed in areas that are more open and sought cover in sites with similar sighting distance as passive bears after being disturbed.

### How did the bears behave when approached?

The bears showed a varied set of behaviours when approached. The majority of the bears left before we passed them, although some bears left and then came back towards the observers before leaving the area. Others remained until we passed before leaving, or simply remained in the area even after the approach. None of these behaviours should be considered abnormal.

We found that the younger bears moved away more often when approached than older, but this difference decreased during the berry season (Table 1). A previous study found that bears chose daybeds with more horizontal vegetation cover during the berry season than the pre-berry season [57]. This might indicate that the bears respond to the increased human activity during autumn (berry pickers, hunters etc) by choosing sites with more cover, and our results show that the bears are more easily disturbed during the berry season.

Grizzly bears' (also *U. arctos*) level of reaction to people has previously been found to not be influenced by distance (closer or further away than 150 m) when in cover [17]. We usually came closer to the bear than 150 m, but also did not find that the distance to the bear influenced whether the bear left or not.

One way to identify disturbance is using a flight response [58], i.e. as a quantitative measurement of a response defined as “the distance to which a person can approach a wild animal without causing it to flee” [59]. Our finding that the bears left at a greater distance from the observers when there was less cover in the initial site (Table 2, Fig. 1), suggests that the bears made a context-dependent decision of when to leave [58]. Escape theory predicts that prey will determine their behaviour based on the behaviour of the predator, and a change in behaviour of the prey will occur when the risk of remaining exceeds the cost of leaving [60], [61]. The cost connected to leaving when approached by humans includes the loss of benefits achieved by continued foraging or resting, the energetic cost caused by leaving the site, and the cost of being detected. If the animal regards itself as well hidden, the benefit of leaving will occur at a shorter distance to the observer than if the animal is in open habitat, hence the animal should leave sooner in an open habitat [60]. Similar results to ours have also been documented in Eurasian lynx [62] and grizzly bears [16], [63].

Another explanation for why bears remained longer at initial sites with more horizontal vegetation cover could be that the cover concealed scents to a certain degree and reduced noise from the observers, and hence delayed the bear's detection of the observers. Bears have an excellent sense of smell [64], and during our approaches, we made sure that the wind blew 90° in relation to our track, i.e. from us towards the bear when we passed it. We simulated hikers by behaving like them during the approaches, regarding the speed of the approach, and the noises we made.

We also found that active bears had a longer FID than passive bears (Table 2, Fig. 2). It is possible that active bears are more vigilant than passive bears, and when the bears already were active, the inclination to change behaviour and start moving away from the observers was probably higher than when the bears were passive. This pattern has been reported in desert bighorn sheep (*Ovis canadensis* Shaw), which were more likely to flee from human disturbance when moving or standing, than when feeding or bedding [65].

Younger bears left the initial site more often than older bears (Table 1), and the younger bears left at a greater distance from the observers than older individuals (Table 2, Fig. 2). We suggest that this could be because young bears are less experienced. Though adult female grizzly bears have been found to be the most risk-averse category and female grizzly bears were normally found further from vehicles, noise, and paved roads than males [18], we did not detect any difference between the sexes in any of our analyses. These findings do not necessarily contradict each other. As mentioned earlier, hunting can cause individuals to become more wary by removing bold animals. As there is no hunting selection for sex in Sweden [66], we suggest that the sexes experience risk from humans in the same way. Hence, there is no difference in wariness and behaviour towards human encounters, even though females might choose habitats further from vehicles, roads and noise when they have the opportunity to choose. We approached the bears in habitats where they were usually not close to humans, hence the exposure to humans was not chosen by the bear itself and the reaction towards a human encounter could be based on the amount of previous experience. We did not detect animals of either sex more often, stressing that boldness did not vary by sex.

It is important to note that FID does not necessarily reflect the entire impact of human disturbance [67]. If a disturbance is great enough, it can cause an extra cost that can influence growth, health, and reproductive fitness [68]. An animal might detect a predator long before it decides to leave [60], and the bears probably reacted internally before reacting in a way that we could record by a change in GPS positions, making it hard to detect when the animal actually reacted initially [69]. A more accurate way to measure the reaction might be by using physiological measurements, such as heart rate [68], [58]. Heart rates of kittiwakes (*Rissa tridactyla* L.) and European shag (*Phalacrocorax aristotelis* L.) increased by 50% when exposed to potentially threatening stimulus, indicating that the birds could be distressed even when there were no visible changes in behaviour [58].

### Management implications

Our findings support an earlier conclusion that the Scandinavian brown bear normally is not aggressive [31]. Human fear can negatively affect the acceptance of bears and other carnivores, and it is important that people receive information about the bears' normal behaviour in order to feel safe when using the outdoors. Our results can contribute to educational material where people can obtain information about the normal behaviour of solitary adult bears, how to behave if they encounter them, and what generally to expect when hiking in bear habitat. Such information would be useful both in areas with an established brown bear population, and in areas where the bears are re-establishing.

Our findings document how solitary adult Scandinavian brown bears normally behave towards humans on foot in the forest. The probability that people will encounter a bear in Scandinavia is small, because the bears occur in low densities, the daytime habitat they choose is normally too dense for hiking, and because the bears normally are wary and avoid confrontations with humans if possible. Even though there seems to be great variation in the bears' reactions towards human disturbance at close range, most bears left the area before the observers passed the bear's initial site. Crucially, none of the bears behaved aggressively towards the observers.

## Supporting Information

Table S1 List of candidate and selected models (lowest AIC value) for remaining or leaving the initial site, and the flight initiation distance (FID) for brown bears when approached by humans on foot in central Sweden in 2006–2009, respectively. We show AIC values, differences in AIC values between the selected model and each candidate model (ΔAIC), and AIC weights (*Wi*).
(DOCX)
