# Supplementary material for: Behaviour of Solitary Adult Scandinavian Brown Bears (Ursus arctos) when Approached by Humans on Foot
Source: PLoS One. 2012 Feb 20;7(2):e31699. doi: 10.1371/journal.pone.0031699 (PMC3282762; doi:10.1371/journal.pone.0031699)
Supplement: Table S1 — List of candidate and selected models (lowest AIC value) for remaining or leaving the initial site, and the flight initiation distance (FID) for brown bears when approached by humans on foot in central Sweden in 2006–2009, respectively. We show AIC values, differences in AIC values between the selected model and each candidate model (ΔAIC), and AIC weights (Wi). (DOCX) [file pone.0031699.s001.docx]

**Table S1.** **List of candidate and selected models**

List of candidate and selected models (lowest AIC value) for remaining or leaving the initial site, and the flight initiation distance (FID) for brown bears when approached by humans on foot in central Sweden in 2006-2009, respectively. We show AIC values, differences in AIC values between the selected model and each candidate model (ΔAIC), and AIC weights (*Wi*).

| **Response variable: remained (0) or left (1)^a^** | **AIC** | **ΔAIC** | ***Wi*** |
| --- | --- | --- | --- |
| Age+Sex+SD+Season+Wind+Observers+Age*Season+Sex*SD+SD*Season | 121.50 | 0.00 | 0.49 |
| Age+Sex+SD+AP+Season+Wind+Observers+Age*Season+Sex*SD+SD*Season | 123.40 | 1.90 | 0.19 |
| Age+Sex+SD+AP+Season+Wind+Observers+Age*AP+Age*Season+Sex*SD+SD*Season | 124.80 | 3.30 | 0.09 |
| Age+Sex+SD+AP+Season+Obsis+Carcass+Wind+Observers+Age*SD+Age*AP+Age*Season+Sex*SD+SD*AP+SD*Season | 126.10 | 4.60 | 0.05 |
| Age+Sex+SD+AP+Season+Carcass+Wind+Observers+Age*AP+Age*Season+Sex*SD+SD*Season | 126.30 | 4.80 | 0.04 |
| Age+Sex+SD+Season+Observers+Age*Season+Sex*SD | 127.40 | 5.90 | 0.03 |
| Age+Sex+SD+AP+Season+Carcass+Wind+Observers+Age*AP+Age*Season+Sex*SD+SD*AP+SD*Season | 128.00 | 6.50 | 0.02 |
| Age+Sex+SD+AP+Season+Obsis+Carcass+Wind+Observers+Age*SD+Age*AP+Age*Season+Sex*SD+Sex*Season+SD*AP+SD*Season | 128.10 | 6.60 | 0.02 |
| Age+SD+Season+Age*Season | 128.40 | 6.90 | 0.02 |
| Age+Sex+SD+Season+Observers+Age*Season+Sex*SD+SD*Season | 128.60 | 7.10 | 0.01 |
| Age+Sex+SD+Season+Age*Season+Sex*SD | 128.90 | 7.40 | 0.01 |
| Age+Sex+SD+Season+Age*Season | 129.10 | 7.60 | 0.01 |
| Age+Sex+SD+AP+Season+Carcass+Wind+Observers+Age*SD+Age*AP+Age*Season+Sex*SD+SD*AP+SD*Season | 129.80 | 8.30 | 0.01 |
| Age+Sex+SD+AP+Season+Obsis+Carcass+Wind+Observers+Age*SD+Age*AP+Age*Season+Sex*SD+Sex*AP+Sex*Season+SD*AP+SD*Season | 130.10 | 8.60 | 0.01 |
| Age+Season+Age*Season | 132.00 | 10.50 | 0.00 |
| Age+Season | 133.30 | 11.80 | 0.00 |
| Season | 146.00 | 24.50 | 0.00 |
| **Response variable: flight initiation distance (FID) (log distance)^a^** |  |  |  |
| Age+SD+AP | 131.20 | 0.00 | 0.52 |
| Age+SD | 132.40 | 1.20 | 0.29 |
| Age+Sex+SD+AP | 134.70 | 3.50 | 0.09 |
| Age+Sex+SD+AP+Wind+Age*AP+Sex*AP+SD*AP | 136.10 | 4.90 | 0.04 |
| Age+Sex+SD+AP+Sex*AP | 136.10 | 4.90 | 0.04 |
| Age+Sex+SD+AP+Season+Wind+Age*AP+Sex*AP+SD*AP | 139.30 | 8.10 | 0.01 |
| Age+Sex+SD+AP+Age*AP+Sex*AP | 142.00 | 10.80 | <0.01 |
| Age+Sex+SD+AP+Season+Wind+Observers+Age*AP+Sex*AP+SD*AP | 144.00 | 12.80 | <0.01 |
| Age+Sex+SD+AP+Age*AP+Sex*AP+SD*AP | 148.50 | 17.30 | <0.01 |
| SD | 148.60 | 17.40 | <0.01 |
| Age+Sex+SD+AP+Season+Wind+Observers+Age*AP+Sex*SD+Sex*AP+SD*AP | 151.70 | 20.50 | <0.01 |
| Age+Sex+SD+AP+Season+Wind+Observers+Age*SD+Age*AP+Sex*SD+Sex*AP+SD*AP | 163.90 | 32.70 | <0.01 |
| Age+Sex+SD+AP+Season+Wind+Observers+Age*SD+Age*AP+Age*Season+Sex*SD+Sex*AP+SD*AP | 170.20 | 39.00 | <0.01 |
| Age+Sex+SD+AP+Season+Wind+Observers+Age*SD+Age*AP+Age*Season+Sex*SD+Sex*AP+SD*AP+SD*Season | 177.40 | 46.20 | <0.01 |
| Age+Sex+SD+AP+Season+Wind+Observers+Age*SD+Age*AP+Age*Season+Sex*SD+Sex*AP+Sex*Season+SD*AP+SD*Season | 179.20 | 48.00 | <0.01 |
| Age+Sex+SD+AP+Season+Carcass+Wind+Observers+Age*SD+Age*AP+Age*Season+Sex*SD+Sex*AP+Sex*Season+SD*AP+SD*Season | 180.70 | 49.50 | <0.01 |

^a^ *SD* cover (sighting distance in the initial site); *AP* activity of the bear (passive = 0. active = 1); *Season* (pre-berry = 0. berry = 1); *Obsis* minimum distance between observer and initial site (only in the binomial model); *Carcass* carcass present at initial site; *Wind* wind strength near bear; *Observers* number of observer
